# Preclinical Efficacy of a PARP-1 Targeted Auger-Emitting Radionuclide in Prostate Cancer

**DOI:** 10.3390/ijms24043083

**Published:** 2023-02-04

**Authors:** Sreeja Sreekumar, Dong Zhou, Cedric Mpoy, Elsa Schenk, Jalen Scott, Jeffrey M. Arbeit, Jinbin Xu, Buck E. Rogers

**Affiliations:** 1 Department of Radiation Oncology, Washington University School of Medicine, St. Louis, MO 63110, USA; 2 Department of Radiology, Washington University School of Medicine, St. Louis, MO 63110, USA; 3 Department of Surgery, Washington University School of Medicine, St. Louis, MO 63110, USA

**Keywords:** Auger emitters, PARP inhibitor, prostate cancer, radionuclide therapy

## Abstract

There is an unmet need for better therapeutic strategies for advanced prostate cancer. Poly (ADP-ribose) polymerase-1 (PARP-1) is a chromatin-binding DNA repair enzyme overexpressed in prostate cancer. This study evaluates whether PARP-1, on account of its proximity to the cell’s DNA, would be a good target for delivering high-linear energy transfer Auger radiation to induce lethal DNA damage in prostate cancer cells. We analyzed the correlation between PARP-1 expression and Gleason score in a prostate cancer tissue microarray. A radio-brominated Auger emitting inhibitor ([^77^Br]Br-WC-DZ) targeting PARP-1 was synthesized. The ability of [^77^Br]Br-WC-DZ to induce cytotoxicity and DNA damage was assessed in vitro. The antitumor efficacy of [^77^Br]Br-WC-DZ was investigated in prostate cancer xenograft models. PARP-1 expression was found to be positively correlated with the Gleason score, thus making it an attractive target for Auger therapy in advanced diseases. The Auger emitter, [^77^Br]Br-WC-DZ, induced DNA damage, G2-M cell cycle phase arrest, and cytotoxicity in PC-3 and IGR-CaP1 prostate cancer cells. A single dose of [^77^Br]Br-WC-DZ inhibited the growth of prostate cancer xenografts and improved the survival of tumor-bearing mice. Our studies establish the fact that PARP-1 targeting Auger emitters could have therapeutic implications in advanced prostate cancer and provides a strong rationale for future clinical investigation.

## 1. Introduction

Prostate cancer is one of the most diagnosed cancers in men worldwide and the second leading cause of cancer deaths in the USA [1,2]. Although patients with non-metastatic prostate cancer at initial diagnosis have a high five-year survival rate, patients who present with or progress to castration resistance and metastases have a poor prognosis with a survival rate nearing 30% [3,4]. Despite the recent therapeutic advances to improve overall survival, including second-generation anti-androgen therapy, immunotherapy, chemotherapy, and radiopharmaceuticals targeting bone, a castration-resistant disease usually represents the lethal stage of advanced prostate cancer [5,6,7,8,9,10,11,12,13,14,15,16]. Hence, there remains a critical need for effective therapeutic strategies to improve the clinical outcomes of patients with advanced prostate cancer. 

Targeting DNA damage repair pathways for cancer therapy has gained much attention over the past decades. Poly (ADP-ribose) polymerase-1 (PARP-1) is a ubiquitous nuclear enzyme that binds DNA and facilitates single-strand break repair [17,18,19]. In response to DNA damage, PARP-1 gets activated and catalyzes the transfer of ADP-ribose from NAD+ onto PARP itself and other protein substrates [20]. Multiple ADP-ribose moieties are sequentially added by PARP-1 forming poly(ADP)ribose which facilitates the repair of damaged DNA [21]. PARP-1 inhibitors compete with NAD+ for the catalytically active site of PARP-1, preventing the synthesis of poly(ADP)ribose, thereby inhibiting DNA repair and leading to cell death [22]. PARP-1 inhibition has been pursued as a therapeutic choice for cancers deficient in homologous recombination repair (HRR) mechanisms because of its ability to induce synthetic lethality [18,23]. PARP-1 is one of the most abundantly expressed nuclear proteins and is found to be overexpressed in the prostate cancer [24,25]. FDA has recently approved the PARP inhibitors rucaparib and olaparib for metastatic castration-resistant prostate cancer (mCRPC) patients with *BRCA1/2* and HRR mutations [26,27]. Patients harboring non-*BRCA* HRR gene mutations experienced inferior outcomes to PARP-1 inhibitor monotherapy compared to those with *BRCA1/2* mutations [27,28,29]. The prevalence of *BRCA1/2* mutations in metastatic prostate cancer patients is around 6.2% [30,31]. That raises the question as to whether and how PARP-1 inhibition therapy can be extended to advanced prostate cancer patients lacking *BRCA1/2* mutations.

Radiolabeled PARP inhibitors have recently been proposed for the imaging and therapy of PARP-expressing cancers with and without HRR mutations [32]. PARP-1 targeted tracers are being evaluated for positron emission tomography (PET) or single photon emission computed tomography (SPECT) imaging of tumors [32,33]. Radio-fluorinated PARP-1 tracers ^18^F-PARPi and 18F-FluorThanatrace (^18^F-FTT) have advanced to PET imaging Phase I clinical trials [34,35]. Riad and coworkers have shown that ovarian tumors in a xenograft mouse model can be imaged by SPECT with PARP theranostic, [^123^I]KX1 [36]. Salinas et al. have reported the utility of radio-iodinated inhibitor ^131/124^I_2_-PARPi as potential PET/SPECT tracers in glioblastoma models [37]. The beta-emitting PARP inhibitor ^131^I-PARPi was found to be effective in extending the overall survival of a mouse model of the glioblastoma [38]. However, ^131^I is a beta-emitter with low linear energy transfer (LET) and is less effective in inducing sufficient DNA damage and cytotoxicity compared to a high-LET radiation [39]. Hence, high-LET alpha and Auger-emitting PARP-1 inhibitors targeting cancer cells should have even greater therapeutic efficacy. Preclinical studies have demonstrated that PARP-1 targeting alpha therapy ([^211^At]MM4) is beneficial in neuroblastoma and augments PD-1 immune checkpoint blockade in the glioblastoma [40,41]. Compared to alpha emitters, Auger-emitters deposit energy over a short range (<100 nm vs. 40–100 µm for alpha), resulting in cancer cell death, especially when the radioligand is incorporated directly into the DNA [42,43,44,45]. PARP-1, owing to its nuclear localization and DNA binding ability, is an excellent candidate for radiolabeling with Auger-emitting radioisotopes. Pirovano et al. demonstrated that an Auger-emitting PARP-1 inhibitor (^123^I-MAPi) improved the survival of mice with glioblastoma [46]. Wilson and his coworkers reported the utility of ^123^I-MAPi in p53 mutated colorectal cancer tumor models [47]. In a recent in vitro study, the Auger emitting PARP-1 inhibitor [^125^I]-PARPi-01 alone or in combination with lipophilic doxorubicin displayed a significant therapeutic response in triple-negative breast cancer [48]. A radioiodinated Auger emitter targeting PSMA, ^125^I-DCIBzL, was found to be effective in delaying the development of metastatic prostate cancer and conferred a survival advantage in a micrometastatic model of the disease [49]. However, to date, no study has investigated the efficacy of a PARP-1 targeting Auger emitter in prostate cancer. 

In the current study, our approach is to target PARP-1 in prostate cancer cells with a PARP-1 inhibitor (WC-DZ-Br) labeled with the Auger emitter bromine-77 ([^77^Br]Br-WC-DZ). Bromine-77 has a half-life of 57.0 h and compared to the frequently used radio-iodinated Auger emitters, it has the advantages that the C–Br bond is more stable, does not accumulate in the thyroid, and offers a more diffuse bio-distribution [50]. The backbone of [^77^Br]Br-WC-DZ is rucaparib, the PARP inhibitor from which radioligands, such as [^18^F]FTT, [^125^I]KX1, and [^211^At]MM4, have been derived [32,51]. We have previously reported the efficacy of a novel rucaparib derivative [^18^F]WC-DZ-F in the PET imaging [35]. In this study, we synthesized ^77^Br]Br-WC-DZ and evaluated the efficacy of the radioligand in prostate cancer cell lines and in vivo subcutaneous models of prostate cancer. Treating prostate cancer in xenograft-tumor-bearing mice with a single dose of [^77^Br]Br-WC-DZ significantly inhibited tumor growth. We also confirmed that [^77^Br]Br-WC-DZ offers a survival advantage to in vivo preclinical prostate cancer models. Our findings, for the first time, illustrated the potential of Auger emitters in prostate cancer therapy and provided the incentive for additional studies.

## 2. Results

### 2.1. PARP-1 Expression in Prostate Cancer and Correlation with Gleason Score 

**Figure 1:**
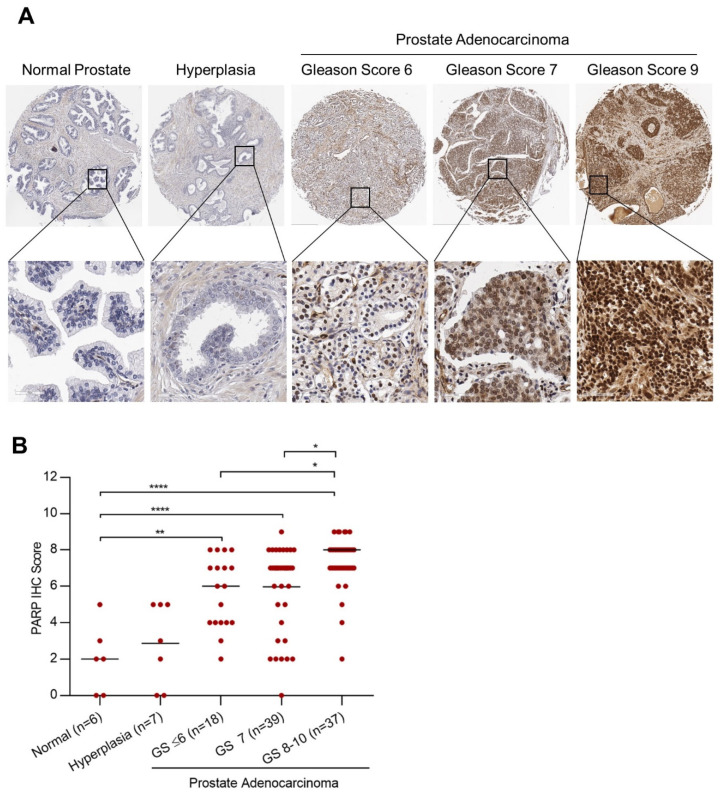
Increase in PARP-1 protein expression with Gleason grade in prostate cancer tissues. (**A**), immunohistochemistry (IHC) staining of PARP-1 in normal human prostate, prostate hyperplasia, and prostate cancer cases graded based on Gleason score in a tissue microarray (TMA). Representative photomicrographs at 4× (top panel) and 40× (lower panel) magnification are shown. (**B**), IHC scoring of PARP-1 expression in the TMA cores. Scatter plots showing the distribution of PARP IHC scores. IHC score is calculated as the sum of the PARP-1 positive proportion (0 = no positive tumor cells; 1 = <1%; 2 = 1–10%; 3 =11–33%; 4 = 34–66%; 5 = 67–100%) and the staining intensity (0, no staining; 1, weak; 2, moderate; 3, strong; 4, very strong) for a possible total score of nine. * *p <* 0.05, ** *p <* 0.01 and **** *p <* 0.0001.

Gleason score is the most widely used prostate cancer grading system and is an effective prognostic predictor in prostate cancer patients [52,53]. We evaluated the expression of PARP-1 in a prostate cancer TMA with normal prostate tissue (n = 6), prostate hyperplasia (n = 7), and prostatic adenocarcinoma (n = 94) graded based on Gleason score ranging from 6 to 10. PARP-1 expression was found to be elevated in prostate adenocarcinoma compared to normal prostate tissue (normal vs. Gleason score ≤ 6: *p* = 0.0011; normal vs. Gleason score 7: *p <* 0.0001; normal vs. Gleason score 8–10: *p* = <0.0001) and prostatic hyperplasia (hyperplasia vs. Gleason score ≤ 6: *p* = 0.0152; hyperplasia vs. Gleason score 7: *p* = 0.0016; hyperplasia vs. Gleason score 8–10: *p* ≤ 0.0001) (Figure 1A,B). The Spearman correlation coefficient was computed to identify the association between PARP-1 expression and Gleason grades. PARP-1 exhibited a statistically significant correlation with the Gleason score (r = 0.35, *p* < 0.0006), displaying an increase in PARP-1 expression with corresponding increases in Gleason score (Gleason score ≤ 6 vs. 8–10: *p* = 0.0312; Gleason score 7 vs. 8–10: *p* = 0.0251). Our data indicate that patients with highly aggressive tumors with high Gleason scores and those with a predicted poor prognosis have elevated PARP-1, making them ideal candidates for PARP-1-targeted radiotherapeutics.

### 2.2. Synthesis, Purification, and PARP Binding Affinity of [^77^Br]Br-WC-DZ 

The radiochemical purity of the synthesized [^77^Br]Br-WC-DZ was greater than 99%, and the molar activity was 21,000 ± 4900 mCi/µmol (n = 12). This high specific activity is ideal for targeted radiotherapy, especially for a saturable target, such as PARP-1, resulting in highly efficient radiation delivery [54]. In this study, we included PC-3, the commonly used in vitro model of CRPC derived from bone metastasis, and IGR-CaP1, a more recently developed cell line that produces completely penetrant bone, liver, and brain metastasis in mice emulating aggressive end-stage prostate cancer [55,56,57]. Both cell lines are androgen-receptor-negative [58]. We have confirmed the PARP-1 expression in PC-3 and IGR-CaP1 cells by immunoblot analysis (Figure S3A). The [^77^Br]Br-WC-DZ displayed high binding affinity (*K*_d_ = 0.58 ± 0.25 nM) for PARP-1 and low non-specific binding, as determined by saturation binding studies in PC-3 tumor xenografts (Figure S3B). To confirm whether the uptake of [^77^Br]Br-WC-DZ in PC-3 and IGR-CaP1 is PARP-1 dependent, the radioligand uptake in these cells in the presence of molar excess of a non-radiolabeled PARP-1 inhibitor olaparib (50 µM) was evaluated. The uptake of [^77^Br]Br-WC-DZ was blocked to background levels in the presence of olaparib in both cell lines, confirming that it is PARP-1-dependent (Figure S3C).

### 2.3. The [^77^Br]Br-WC-DZ-Induced Cytotoxicity and Inhibited the Colony Formation of Prostate Cancer Cells

**Figure 2:**
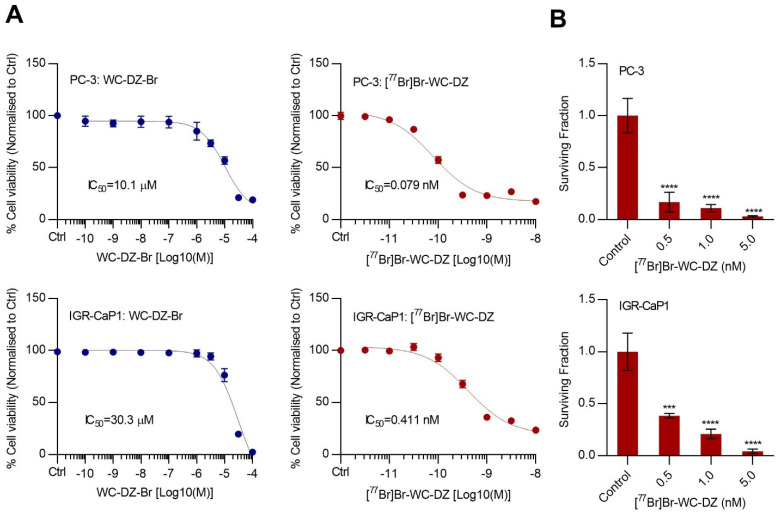
The [^77^Br]Br-WC-DZ induces cytotoxicity and decreases the colony-forming ability of prostate cancer cell lines. (**A**), PC-3 and IGR -CaP1 cells were treated with increasing doses of WC-DZ-Br (10^−10^ to 10^−4^ M) or [^77^Br]Br-WC-DZ (10^−11.5^ to 10^−8^ M) in biological replicates of five for 120 h. Cell viability was determined using an MTS assay. Graph represents a percentage (%) mean cell viability normalized to control ± SD. (**B**), the effect of [^77^Br]Br-WC-DZ on prostate cancer cell survival. PC-3 and IGR-CaP1 cells were treated with [^77^Br]Br-WC-DZ (0.5, 1, and 5 nM) in biological replicates of three for 3h, washed, and the ability of the cells to form colonies was assessed after 14 days in a clonogenic survival assay. *** *p <* 0.01 and **** *p <* 0.0001 compared to the control.

The in vitro cytotoxic effect of [^77^Br]Br-WC-DZ in PC-3 and IGR-CaP1 cell lines was evaluated. In both cell lines, [^77^Br]Br-WC-DZ-induced dose-dependent cytotoxicity with sub-nanomolar IC_50_ concentrations of 0.079 nM in PC-3 and 0.411 nM in IGR-CaP1 (Figure 2A). The non-radiolabeled inhibitor WC-DZ-Br was not effective at killing the cells at these concentrations and displayed IC_50_ values in micromolar ranges of 10.1 µM in PC-3 and 30.3 µM in IGR-CaP1 (Figure 2A)_._ Additionally, the PARP-1 inhibitor rucaparib, which is the backbone for the Auger emitter, showed a negligible cytotoxic effect in PC-3 (IC_50_ = 3.68 µM) and IGR-CaP1 (IC_50_ = 18.4 µM) cells at this sub-nanomolar concentration range (Figure S4). Altogether, these findings suggest that the cytotoxic potential of [^77^Br]Br-WC-DZ is due to the sensitivity of the cells to the Auger radiation that is delivered to the target DNA by the PARP-1 inhibitor.

PC-3 and IGR-CaP1 cells were treated with varying doses (0.5, 1.0, and 5.0 nM) of [^77^Br]Br-WC-DZ, and clonogenic survival was assessed. We observed a significant decrease (*p <* 0.001 in all doses tested) in the surviving fraction compared to the control in both cell lines (Figure 2B). The surviving fraction values at the lowest concentration (0.5 nM) were decreased to 0.25 and 0.45 in PC-3 and IGR-CaP1, respectively (Figure 2B). PC-3 cells were more sensitive to [^77^Br]Br-WC-DZ compared to IGR-CaP1. The effect was found to be dose-dependent in IGR-CaP1 (*p <* 0.05; 0.5 nM vs. 5 nM) but not in PC-3 cells. 

### 2.4. [^77^Br]Br-WC-DZ Induce PARP-1 Dependent DNA Damage

**Figure 3:**
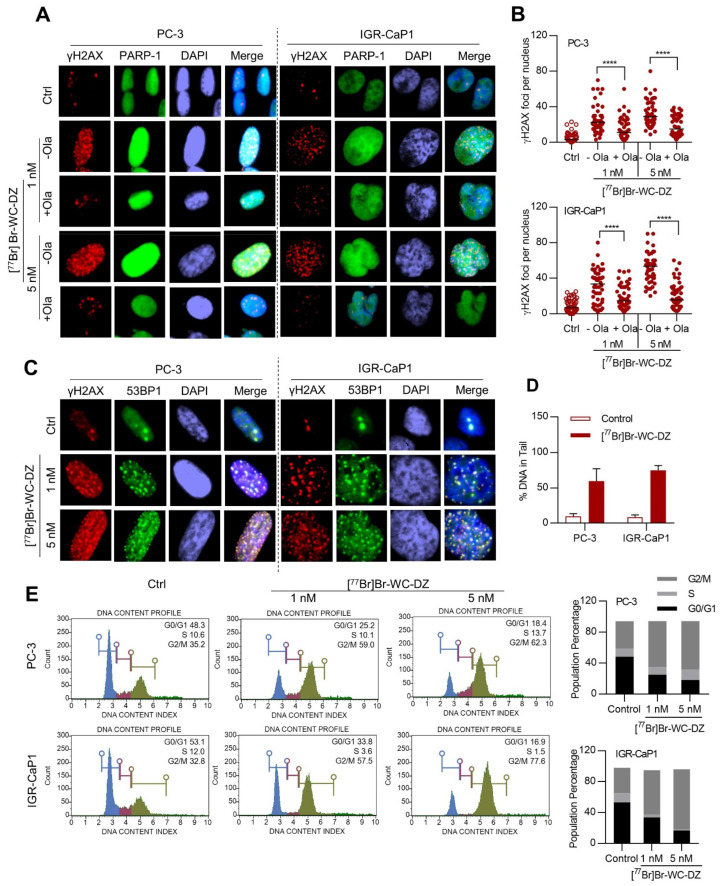
The [^77^Br]Br-WC-DZ-induced DNA damage arrest of the cells at the G2/M checkpoint. (**A**,**C**), immunofluorescence of γH2AX, PARP-1, and 53BP1 in PC-3 and IGR-CaP1 cells after 24-h treatment with [^77^Br]Br-WC-DZ (1 nM/5 nM) in the presence and absence of olaparib (20 µM). Representative immunofluorescence images are shown. The original images were captured at 100× (PC-3) and 63× (IGR-CaP1) magnification with oil immersion. (**B**), γH2Ax foci counts per nucleus in the cells treated with [^77^Br]Br-WC-DZ. γH2Ax foci were counted on at least 50 cells per treatment, and results are depicted as dot plot distribution values (the median is also reported for each sample) **** *p* < 0.0001. (**D**), DNA damage (% DNA in the tail) measured with the Comet assay after treatment with [^77^Br]Br-WC-DZ (5 nM) for 4 h. (**E**), cell-cycle analysis of [^77^Br]Br-WC-DZ treated cells showing accumulation at the G2/M phase of the cell cycle. Histogram depicting the percentage of accumulated cells in the G0/G1 (blue), S (purple) and G2/M phase (green) of the cell cycle.

To elucidate the mechanism of cytotoxicity induced by [^77^Br]Br-WC-DZ, we performed immunofluorescence analysis of phosphorylated histone 2A family member X (γH2AX) and tumor suppressor p53 binding protein 1 (53BP1) DNA repair nuclear foci, which are markers of the radiation-induced DNA damage [59]. In both PC-3 and IGR-CaP1 cells, [^77^Br]Br-WC-DZ caused a significant (*p <* 0.0001) increase in γH2AX foci formation at both 1 and 5 nM concentrations (Figure 3A,B). The increase was time-dependent in both cell lines and dose-dependent (*p <* 0.0001) in IGR-CaP1 (Figure 3A,B and Figure S5A,B). There was also an increase in PARP-1 expression after treatment with [^77^Br]Br-WC-DZ for 24 h (Figure 3A). In blocking experiments when cells were co-treated with [^77^Br]Br-WC-DZ and an excess of PARP inhibitor olaparib, γH2AX foci formation was significantly decreased (*p <* 0.0001), suggesting that the DNA damage is specific to [^77^Br]Br-WC-DZ binding to PARP-1 (Figure 3A,B). The γH2AX foci were found to be co-localizing with 53BP1, which is an index of radiation-induced DNA damage (Figure 3C). To further analyze the residual DNA damage after treatment with [^77^Br]Br-WC-DZ, cells were treated with the Auger emitter for 1 h, media was washed out, and γH2AX foci formation was assessed. There was a significant increase in γH2AX foci formation in both PC-3 and IGR-CaP1 (*p <* 0.0001) cells compared to the control (Figure S5C,D). 

A comet assay was performed in PC-3 and IGR-CaP1 cells treated with [^77^Br]Br-WC-DZ. The amount of DNA damage is expressed as the % of DNA in the tail (olive moment). Upon treatment with [^77^Br]Br-WC-DZ for 4 h, the % of DNA in the tail increased from 9.71 ± 0.88 to 59.45 ± 4.08% in PC-3 and 8.69 ± 0.78 to 74.69 ± 1.58% in IGR-CaP1 cells (Figure 3D). To assess the sensitivity of the cells to the DNA damage induced by the Auger emitter, cell cycle phase distribution (G0/G1, S, and G2/M) was analyzed. The [^77^Br]Br-WC-DZ induced the accumulation of PC-3 and IGR-CaP1 cells in the G2/M phase of the cell cycle with a concomitant decrease in the population of cells in the G0/G1 and S phase (Figure 3E). The effect was found to be dose-dependent in IGR-CaP1 but not PC-3 cells, consistent with what was noticed in the survival assays and γH2AX foci formation. The lack of dose-dependent effects in PC-3 could be attributed to the saturation of the available PARP-1 target at the lower concentrations of the radioligand. Overall, the experiments confirmed that [^77^Br]Br-WC-DZ induced DNA double-strand breaks (DSBs), leading to the arrest of the cells at the G2/M checkpoint and, thereby, cytotoxicity.

### 2.5. Biodistribution of [^77^Br]Br-WC-DZ in Vivo

**Figure 4:**
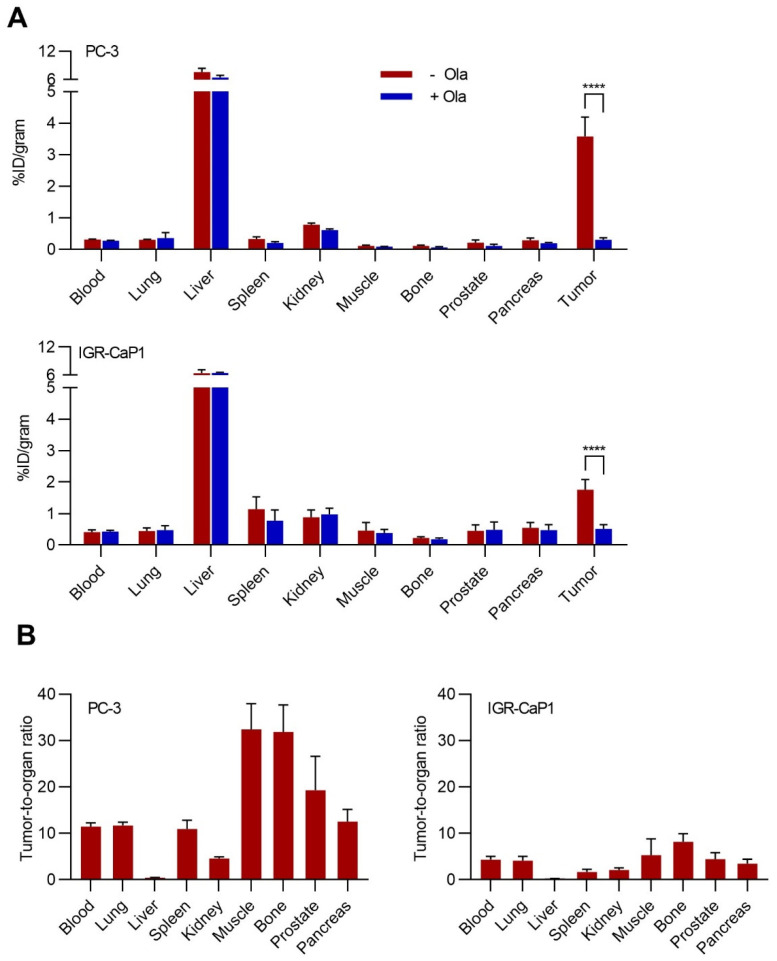
(**A**), biodistribution of [^77^Br]Br-WC-DZ (370 kBq) at 4h (+/− 5 mg/kg bwt olaparib block) in athymic nude mice (n = 5) bearing PC-3 and IGR-CaP1 tumors. The data represent the mean ± SD. **** *p* < 0.0001 (**B**), tumor-to-organ ratios of uptake of [^77^Br]Br-WC-DZ (370 kBq) at 4h in PC-3 ad IGR-CaP1 tumors and selected organs of tumor-bearing mice.

Biodistribution studies of [^77^Br]Br-WC-DZ in the presence and absence of a blocking agent, olaparib, were performed in mice bearing PC-3 and IGR-CaP1 xenografts to confirm selective tumor localization and absence of specific uptake in normal tissues. Both PC-3 and IGR-CaP1 xenograft-bearing mice showed tumor uptake (PC-3, 3.58 ± 0.61 %ID/g; IGR-CaP1, 1.76 ± 0.32 %ID/g) of [^77^Br]Br-WC-DZ at 4 h (Figure 4A). Olaparib significantly reduced the radioactive uptake of both PC-3 (3.58 ± 0.61 vs. 0.31 ± 0.07 %ID/g *p*, 0.0001) and IGR-CaP1 (1.76 ± 0.32 vs. 0.52 ± 0.13 %ID/g *p*, 0.0001) tumors (Figure 4A). Assessment of radioactive distribution patterns in blood and normal organs showed negligible uptake in blood, lung, muscle, bone, prostate, and pancreas (<0.5 %ID/g), slight uptake in the kidney and spleen (<1.2 %ID/g), and highest uptake in the liver (6–7 %ID/g) (Figure S6). High tumor-to-tissue ratios (>2) were observed in both tumor models for all tissues except for the liver (Figure 4B). Olaparib blocking did not significantly reduce the radioactivity uptake in any of the normal organs confirming that its uptake is PARP-1-independent (Figure 4A). Taken together, these findings confirm the in vivo binding specificity and selectivity of [^77^Br]Br-WC-DZ toward PARP-1-expressing tumors.

### 2.6. [^77^Br]Br-WC-DZ Delayed Prostate Cancer Xenograft Tumor Growth

**Figure 5:**
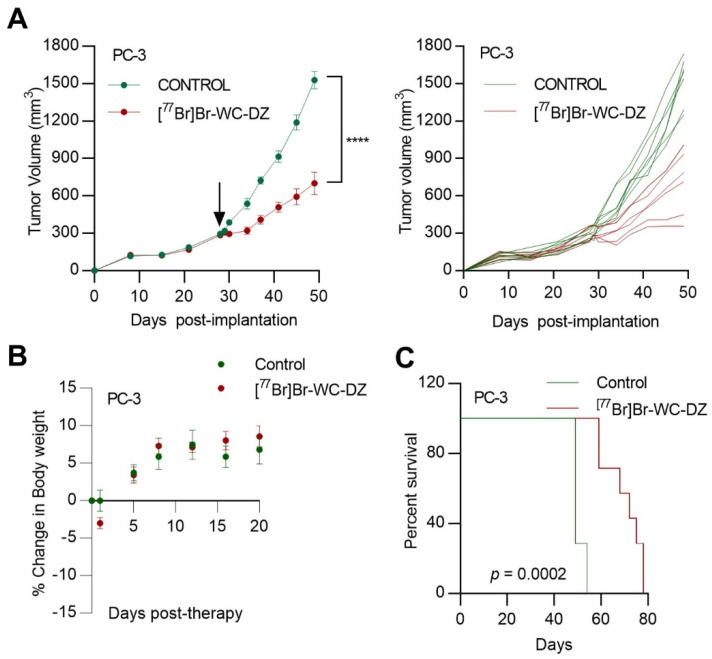
The [^77^Br]Br-WC-DZ-inhibited tumor growth in animal models of prostate cancer. (**A**), mean tumor volume (±SEM) in athymic nude mice (n = 7) after treatment (indicated by black arrow) with 56 MBq of [^77^Br]Br-WC-DZ. Control group received saline. Individual tumor-growth curves for control and [^77^Br]Br-WC-DZ-treated groups are also shown. (**B**), the plots showing the average percent change in body weight of mice treated with control or [^77^Br]Br-WC-DZ. (**C**), Kaplan–Meier survival study of PC-3-tumor-implanted mice showed improved survival of [^77^Br]Br-WC-DZ-treated mice. Statistical significance was determined using a Mantel–Cox log-rank test. **** *p* < 0.0001.

We evaluated the in vivo antitumor efficacy of [^77^Br]Br-WC-DZ in nude mice bearing PC-3 and IGR-CaP1 xenografts. To these ends, *nu/nu* mice bearing PC-3 and IGR-CaP1 tumors received a single intravenous dose of 56 MBq of [^77^Br]Br-WC-DZ. Mice in the control group were administered an equivalent volume of saline. While the animals in the control group showed continued exponential growth of the PC-3 tumors, those treated with [^77^Br]Br-WC-DZ demonstrated a delay in tumor growth (*p* < 0.0001) (Figure 5A). Additionally, the [^77^Br]Br-WC-DZ treated groups did not display any weight loss indicating the lack of radionuclide-induced systemic toxicity (Figure 5B). The [^77^Br]Br-WC-DZ conferred a significant survival advantage in PC-3-tumor-bearing mice, with median survival in the control and treatment group being 49 days versus 72 days (*p* = 0.0002) (Figure 5C). The IGR-CaP1 tumors also showed a delay in tumor growth with [^77^Br]Br-WC-DZ treatment (*p* = 0.0296), and there was a significant survival advantage in treated mice with median survival in control and treatment being 83 days versus 90 days (*p* = 0.0294) (Figure S7). Compared to IGR-CaP1 xenografts, PC-3 tumors responded to [^77^Br]Br-WC-DZ more efficiently and showed more dramatic effects in terms of tumor growth restriction and survival advantage. This finding correlates with the increased uptake of the radioligand in PC-3 tumors, as evidenced in the biodistribution studies (Figure 4). Overall, this study, for the first time, provides evidence that a PARP-1-targeting Auger emitter can offer antitumor effects in in vivo prostate cancer models with minimal toxicity. 

## 3. Discussion

While there are cures available for localized prostate cancer, the advanced disease poses a therapeutic challenge because of castration or chemotherapy resistance, demanding effective curative strategies. Targeted radiotherapy is emerging as a promising treatment modality that efficiently delivers ionizing radiation to cancer cells while minimizing radiation exposure to untargeted cells. Despite the efficacy of the currently used radioligands, including the beta-emitter ^177^Lu-PSMA-617 for mCRPC, there is still a need for more specific targets and radionuclides with improved treatment outcomes and minimal side effects [60,61]. In this study, we are reporting the preclinical evaluation of an Auger-emitting radio-brominated PARP-1 inhibitor, [^77^Br]Br-WC-DZ, in prostate cancer cell lines and in vivo models.

Our TMA analysis corroborated the previous studies that PARP-1 expression is higher in prostate cancer than in normal prostate tissues [24,25,62,63]. Most importantly, our study is the first to markedly correlate the PARP-1 expression with the Gleason score. We have previously reported the utility of PARP-1 radioligands, specifically [^18^F]WC-DZ-F, for PET imaging in the prostate cancer [64]. The Auger emitter [^77^Br]Br-WC-DZ synthesized for this study is an analog of [^18^F] WC-DZ-F, except that fluorine is replaced with bromine. Although Auger therapy has been predominantly studied with ^125^I, the advantages of ^77^Br include its high radiotoxicity, longer half-life, higher theoretical specific activity, and lack of thyroid uptake [65,66]. As far as we know, this is the first study exploring the effectiveness of a ^77^Br linked PARP-1 inhibitor in in vivo xenograft tumor models. 

Cytotoxicity studies confirmed that, on a molar level, [^77^Br]Br-WC-DZ is more potent than non-radiolabeled WC-DZ-Br displaying more than a 10,000-fold difference in IC_50_ values. DNA repair foci analysis in the presence of blocking agents, such as olaparib, revealed that [^77^Br]Br-WC-DZ-induced DNA damage is brought about by its specific binding to PARP-1. Furthermore, we observed an increase in PARP-1 expression with [^77^Br]Br-WC-DZ because of the DNA-damage-induced upregulation of PARP-1. Considering the intact homologous recombination repair genes such as *BRCA1* and *2* in the cell lines tested, the cytotoxic effect is brought about by DNA damage induced by ionizing radiation rather than synthetic lethality by PARP-1 inhibition. Additionally, keeping in mind that patients’ harboring *BRCA1*/*2* or HRR mutations are benefitted from conventional PARP-1 inhibitors, PARP-1 Auger therapy has the advantage that its use can be extended beyond *BRCA1*/*2* defective cancers.

According to some of the earlier works, despite showing potent in vitro cytotoxic activity, PARP-1 inhibitor monotherapy showed limited clinical activity in certain tumor types [67]. Lee et al. have reported that, though a PARP-1-targeted Auger emitter ^125^I-KX1 was highly cytotoxic in vitro, it was predicted to display limited therapeutic efficacy in solid tumor models of neuroblastoma [39]. When administered intratumorally, ^131^I-PARPi elicited a significant reduction in tumor growth and improvement in median survival of the subcutaneous mouse model of the glioblastoma [38]. Similarly, Auger-emitting PARP inhibitor ^123^I-MAPi displayed therapeutic efficacy in glioblastoma models employing a complex convection-enhanced drug delivery system [46]. In the present study, the systemically administered brominated Auger emitter [^77^Br]Br-WC-DZ was effective in suppressing the growth of subcutaneous prostate cancer tumors and offered a survival advantage in tumor-bearing mice. These results could be improved by optimizing the radiation dose administered and by investigating multi-dose fractions. We would be expanding the in vivo studies to micro-metastatic models of prostate cancer.

Though PARP-1 expression levels were similar between the PC-3 and IGR-CaP1 cell lines, PC-3 cells showed increased sensitivity to [^77^Br]Br-WC-DZ in in vitro assays in terms of cytotoxicity, clonogenic survival, and in in vivo xenograft tumor suppression. The differential sensitivity could be attributed to several factors, including cell type, DNA repair capacity, cell cycle phase at the time of exposure, and the microenvironment [68]. PC-3 cells have a homozygous deletion of the DNA repair-associated gene *PTEN* [69]. This could also be contributing to the increased sensitivity of PC-3 cells to [^77^Br]Br-WC-DZ. Further studies are warranted to identify the contributing factors leading to the sensitivity and/or resistance of the cells to [^77^Br]Br-WC-DZ to better understand the clinical outcome.

In conclusion, this study identifies the utility of PARP-1 inhibition as a means for delivering lethal high-LET Auger radiation to the cancer cell’s DNA resulting in tumor-specific DNA damage and cytotoxicity. Radio-brominated Auger emitting PARP-1 inhibitor [^77^Br]Br-WC-DZ could have the potential for clinical translation in advanced prostate cancer and warrants further investigation. Since PARP-1 is overexpressed in a multitude of cancers, this therapeutic approach has the prospective to be extrapolated to other cancers as well.

## 4. Materials and Methods

### 4.1. Cell Lines and Culture Conditions

Human prostate cancer cell line PC-3 was obtained from American Type Culture Collection (Manassas, VA, USA). The cells were cultured in high-glucose Dulbecco’s modified eagle medium (DMEM) supplemented with 10% fetal bovine serum (Gibco, Life Technologies, Carlsbad, CA, USA). The IGR-CaP1 cell line, derived from primary prostate cancer, was kindly provided by Dr. Anne Chauchereau (Prostate Cancer Group, Institut Gustave Roussy, Villejuif, France) [57]. IGR-CaP1 cells were cultured according to the culture conditions described previously [56]. The cell lines were maintained at 37 °C, 5% CO_2_ in a humidified incubator. The cell lines were authenticated by short-tandem repeat (STR) profiling (Arizona Genetics Core, Tucson, AZ, USA). 

### 4.2. PARP-1 Expression in Prostate Cancer Tissue Microarray

The prostate cancer tissue microarray (TMA) was purchased from US Biomax, Inc. (Rockville, MD, USA). The TMA slide was deparaffinized in xylene and rehydrated by serial incubations in graded ethanol and then in distilled water. Antigen retrieval was performed by incubating the slides in pre-boiled citrate buffer, pH 6.0 (#C9999, Sigma-Aldrich, St Louis, MO, USA) in a steamer for 20 min, followed by cooling at room temperature for 15 min. The slides were washed in 1× Wash buffer (#S3006, Agilent Dako, Santa Clara, CA, USA), and endogenous peroxidase was quenched by incubating with Dual Endogenous Enzyme Block (#S2003, Agilent Dako, Santa Clara, CA, USA) for 10 min. The tissue sections were blocked (10% normal goat serum, 45 min) and immunostained with PARP (46D11) rabbit antibody (1:300; #9532, Cell Signaling Technologies, Beverly, MA, USA), followed by ImmPRESS goat anti-rabbit immunoglobulins/HRP (#MP-7451, Vector Laboratories, Burlingame, CA, USA). The color was developed using a 3, 3-diaminobenzidine tetrahydrochloride (DAB) substrate chromogen system (#K3467, Agilent Dako, Santa Clara, CA, USA). The sections were counterstained with hematoxylin, dehydrated with ethanol, cleared in xylene, and mounted using Cytoseal XYL mounting medium (#83124, Thermo Scientific, Waltham, MA, USA). The stained slides were scanned using an AperioCS2 Scanner and visualized and analyzed using Aperio ImageScope software (Leica Biosystems, Newcastle, UK). Immunohistochemical quantification of PARP-1 was calculated as a sum of the percentage of cells with detectable levels of PARP-1 (0 = no positive tumor cells; 1 = <1%; 2 = 1–10%; 3 = 11–33%; 4 = 34–66%; 5 = 67–100%) and the staining intensity (0, no staining; 1, weak; 2, moderate; 3, strong; 4, very strong) with a maximum score of 9. 

### 4.3. Chemistry and Radiochemistry 

Br-WC-DZ and the labeling precursor were synthesized as reported (Patent No WO/2002/044183 [70,71]. The radiosynthesis, purification, and dose preparation of [^77^Br]Br-WC-DZ are detailed in the supplementary methods. The [^77^Br]Bromide was produced in the cyclotron facility of Washington University in Saint Louis [72]. The [^77^Br]Br-WC-DZ was prepared via copper-mediated nucleophilic radiobromination of a boron precursor with [^77^Br]Bromide, a strategy that has been reported previously by us [66]. The [^77^Br]Br-WC-DZ was purified by HPLC (Figures S1 and S2). Cellular uptake of [^77^Br]Br-WC-DZ in PC-3 and IGR-CaP1 cell lines in the presence and absence of molar excess of PARP inhibitor olaparib (50 µM) was studied as reported earlier by us [64].

### 4.4. In Vitro Cytotoxicity

The cytotoxic effect of [^77^Br]Br-WC-DZ on PC-3 and IGR-CaP1 prostate cancer cells was determined using the CellTiter 96 Aqueous One Solution Cell Proliferation kit (Promega, Madison, WI, USA) following the manufacturer’s instructions. Briefly, the cells were seeded at a density of 1000–2000 cells/well in 96-well plates. Approximately 24 h after cell seeding, the cells were treated with varying doses (10^−11.5^ to 10^−8^ M/1.8 KBq/mL–5.7 MBq/mL) of [^77^Br]Br-WC-DZ. After incubating for 120 h, the medium was aspirated, and 100 µL of serum-free media and 20 µL of MTS solution (5 mg/mL solution in PBS) were added into each well; the cells were then incubated for 2 h at 37 °C. The absorbance was measured at 490 nm using VersaMax Microplate Reader (Molecular Devices LLC, San Jose, CA, USA). Experiments were completed in six replicates and were repeated three times. The absorbance of vehicle (0.1% ethanol)-treated control cells was considered 100% survival. The half-maximal inhibitory concentration (IC_50_) values were determined using GraphPad Prism 7.0 software (GraphPad Software, Inc., San Diego, CA, USA). Cytotoxicity experiments with non-radioactive WC-DZ-Br and other PARP inhibitors (10^−10^ to 10^−4^ M) were carried out in a similar manner. 

### 4.5. Clonogenic Assay 

Prostate cancer cells were plated in 6-well plates at a density of 500 cells/well in triplicates, allowed to attach overnight, and treated with [^77^Br]Br-WC-DZ (0.5, 1.0, and 5 nM/0.29, and 0.57 and 2.9 MBq/mL). Vehicle (0.01% ethanol)-treated cells served as control. After incubating for 3 h, the cells were washed twice with medium and cultured for 14 days, with fresh medium added every three days. The colonies were fixed (Acetic acid/methanol; 1:7) and stained with 0.5% crystal violet solution. The colonies with more than 50 cells in each well were counted. Plating efficiency (PE = the number of colonies/the number of seeded cells × 100%) and surviving fraction (SF = the number of colonies formed after treatment/number of cells seeded × PE) were calculated. 

### 4.6. Immunofluorescence

Immunofluorescence studies were performed on cells seeded at a density of 50,000 cells/well on coverslips placed inside the 24-well plates. The cells were incubated with varying doses (1 nM, 5 nM/0.57 MBq/ml, 2.8 MBq/ml) of [^77^Br]Br-WC-DZ in the presence or absence of olaparib (20 µM) for indicated durations. Cells were then fixed in 10% neutral buffered formalin for 10 min, followed by three washes with PBS. The cells were then permeabilized (0.1% Triton-X in PBS for 10 min), followed by blocking with 10% goat serum (45 min). Primary antibodies for γH2AX (#05-636-I, Millipore, Burlington, MA, USA; 1.5 µg/mL) or PARP-1 (46D11, #9532, Cell Signaling Technologies, Beverly, MA, USA; 1 in 1000), or 53BP1 (#NB100-304; Novus Biologicals, LLC, Centennial, CO, USA; 2 µg/mL) were added and incubated at 4 °C overnight. The cells were then incubated with AF488 Goat anti-Rabbit (#A11008) or AF594 Goat anti-Mouse (#A32742) IgG secondary antibodies (Thermo Fisher Scientific, Rockford, IL, USA) for 45 min, washed, and the coverslips were mounted on microscopic slides using ProLong Antifade Mountant (Thermo Fisher Scientific, Rockford, IL, USA) containing DAPI. The images were captured using an inverted fluorescence microscope (Olympus, Tokyo, Japan).

### 4.7. DNA Damage Assessment by Comet Assay

The ability of [^77^Br]Br-WC-DZ to induce DNA damage was measured by comet assay. PC-3 and IGR-CaP1 cells were treated with 5 nM [^77^Br]Br-WC-DZ for 4 h. Cells were harvested, suspended in PBS, mixed with low-melting agarose, and spread on a Trevigen’s CometSlide (Gaithersburg, MD, USA). The slides were placed in lysis buffer, followed by single-cell electrophoresis, and stained with 1X SyberGreen (Thermo Fisher Scientific, Rockford, IL, USA) at RT for 30 min in the dark. The CometSlides were then washed and allowed to dry overnight at RT. Images were taken using Zeiss Axioplan 2 microscope using a 20× objective, and the comet tail length was measured using CometScore software (TriTek Corporation, Sumerduck, VA, USA), and results were expressed as the percent of DNA in the tail (tail intensity).

### 4.8. Cell-Cycle Analysis by Flow Cytometry

The effect of [^77^Br]Br-WC-DZ in altering the cell cycle phases of prostate cancer cells PC-3 and IGR-CaP1 was analyzed using a Muse™ Cell Cycle Kit (Luminex, Austin, TX, USA) according to the manufacturer’s instructions. Briefly, after incubating the cells with [^77^Br]Br-WC-DZ (1 nM and 5 nM) for 24 h, the cells were harvested using trypsin and washed with PBS. The cells were then fixed with 70% ethanol kept at −20 °C. The cells were then washed, pelleted, and incubated with Muse cell cycle reagent for 30 min at room temperature in the dark. The percentage of cells in G0/G1, S, and G2/M phases were assessed in Guava Muse™ Cell Analyzer (Luminex, Austin, TX, USA).

### 4.9. In vivo Biodistribution and Antitumor Activity Studies

The animal experiments were approved by the Institutional Animal Care and Use Committee (IACUC) of Washington University School of Medicine (St Louis, MO, USA). Six-week-old athymic female/male nude mice (nu/nu) were purchased from Charles River Laboratories (Wilmington, MA, USA), and animals were acclimated for 1 week before the experiments. In vivo biodistribution studies were performed at the Washington University Small Animal Imaging Facility. PC-3 or IGR-CaP1 cells (1 × 10^7^ cells in serum-free DMEM) were injected into the right flank of female and male mice, respectively. Once the tumors reached a volume of 100 mm^3^, 370 kBq of [^77^Br]Br-WC-DZ was injected intravenously, and the biodistribution was evaluated at 2 h and 4 h time points (n = 5). In the blocking group, mice were pre-injected with olaparib (5 mg/kg bwt), and biodistribution was evaluated at 4 h. The animals were sacrificed at each time point, their organs were harvested, and radioactivity was measured on a Beckman Gamma-8000 counter. Tumor and organ uptake were analyzed and calculated as a percentage of injected dose per weight of tissue in grams (%ID/g). Tumor-to-organ ratios were calculated for each mouse based on %ID/g. For the in vivo efficiency study, once the tumors reached a mean volume of 175 mm^3^, the mice were divided into two groups. One group received saline, and the other received 56 MBq of [^77^Br]Br-WC-DZ by intravenous injection. Tumor volumes were measured using calipers and calculated using the formula: Volume (mm^3^) = (Length × Width^2^)/2. Tumor volume and body weight were monitored twice a week. 

### 4.10. Statistical Analysis 

Statistical analyses were performed using Graph Pad Prism 7 (San Diego, CA, USA) software. Comparisons between groups were made using an unpaired *t*-test (two groups) or one-way/two-way ANOVA (three or more groups) with Bonferroni multiple comparison test. In vivo efficacy studies were evaluated using Kaplan–Meier survival curves compared with the log-rank (Mantel–Cox) test. *p* values < 0.05 were considered significant and are indicated by asterisks in figures (****, *p* < 0.0001; ***, *p* < 0.001; **, *p* < 0.01; *, *p* < 0.05). 

## Data Availability

Data are provided within the article and in the Supplementary Material.

## Supplementary Materials

The following supporting information can be downloaded at: https://www.mdpi.com/article/10.3390/ijms24043083/s1.
