# Supplementary material for: Preclinical Efficacy of a PARP-1 Targeted Auger-Emitting Radionuclide in Prostate Cancer
Source: Int J Mol Sci. 2023 Feb 4;24(4):3083. doi: 10.3390/ijms24043083 (PMC9967758; doi:10.3390/ijms24043083)
Supplement: Supplementary file 1 [file ijms-24-03083-s001.zip › ijms-2142721-supplementary.pdf]

Supplementary Information

Supplementary Methods

Radiosynthesis and characterization of [^{77}Br]Br-WC-DZ-Br

General Information

All chemicals were obtained from standard commercial sources and used without further purification. All reactions were carried out by standard air-free and moisture-free techniques under an inert atmosphere with dry solvents unless otherwise stated. No carrier added [^{77}Br]bromide was produced by the cyclotron facility in Washington University in Saint Louis and delivered in water (0.5-1 mL). High performance liquid chromatography (HPLC) was performed with an ultraviolet detector and a well-scintillation NaI (Tl) detector and associated electronics for radioactivity detection. Radio-TLC was accomplished using a Bioscan AR-2000 imaging scanner (Bioscan, Inc., Washington, DC). Published methods were used for the synthesis of compound Br-WC-DZ (WO2002044183) and its labeling precursor [1].

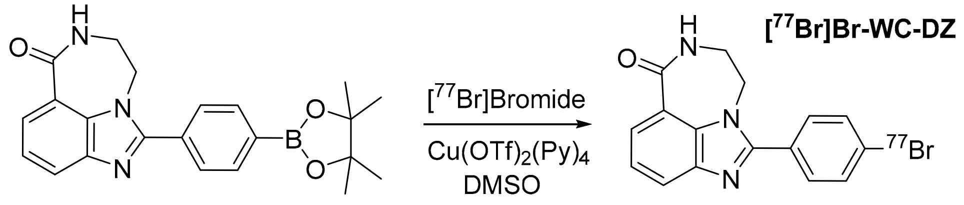

1. **Drying of [^{77}Br]Bromide:** Into a 5 mL Wheaton V vial containing [^{77}Br]bromide in water (0.5-1 ml) a diluted solution of $NH_3 \cdot H_2O$ (1:100, 25 µL) was added, and water was removed under a gentle flow of argon at 110 °C via a charcoal trap. When about 50 µL water was left in the bottom, the vial was removed from heating and the argon flow continued at room temperature until all water was removed.
2. **Radiobromination:** The vial was opened to air to replace the argon atmosphere. Into the dried [^{77}Br]Bromide a solution of the precursor (~0.5 mg) and $Cu(OTf)_2(Py)_4$ (2.5 mg) in DMSO (0.3 mL was added. The reaction vial was vortexed, vented via 18 Ga needle to the air atmosphere and heated at 110 °C. Upon completion of the reaction according to HPLC analysis (10-15 min), the vial was removed from heating and the mixture was diluted with 0.1 % TFA for HPLC purification.
3. **HPLC purification:** The above mixture was injected onto a semi-preparative column (Agilent SB-C18 250×9.4 mm or Phenomenex Luna C18 250×10 mm), eluted with 19.5 % acetonitrile/80.5 % water/0.1 % TFA at a flow rate of 4 mL/min and UV at 250 nm.

4. **Dose preparation:** The radioactive peak of [^{77}Br]Br-WC-DZ was collected and diluted with water (40 mL), and the diluted solution was then passed through a Waters HLB light cartridge to extract [^{77}Br]Br-WC-DZ, which was further rinsed with water and eluted with ethanol. If needed, ethanol was removed under a flow of argon for the final dose of [^{77}Br]Br-DZ-WC in 10% ethanol/saline. The final concentration of ethanol was below 0.1% in *in vitro* experiments. Ethanol vehicle controls (ranging from 0.01 to 0.1%) was included in the *in vitro* studies.

Immunoblot analysis

PC-3 and IGR-CaP1 cells were lysed in RIPA buffer and the protein extracts were resolved by SDS-PAGE and transferred to PVDF membrane. The membranes were probed with PARP-1 antibody (46D11, #9532, Cell Signaling Technologies, USA; 1 in 1000) followed by Horseradish peroxidase-conjugated goat anti-rabbit IgG (#7074, Cell Signaling Technologies, USA; 1 in 2000). Bound antibodies were visualized using enhanced chemiluminescence (Super Signal West Pico; Thermo Fisher Scientific, Rockford, IL, USA).

Supplementary Figures

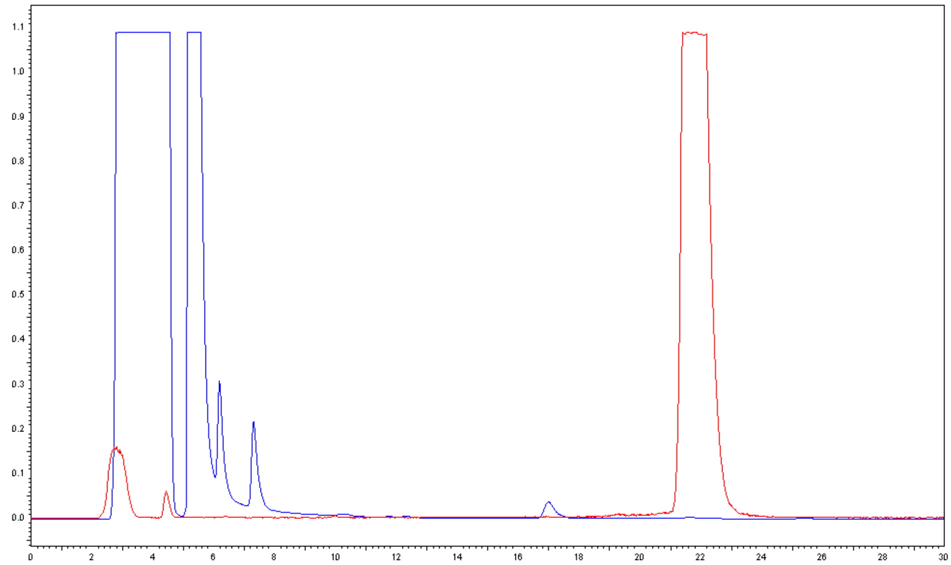

Figure S1. Semi-preparative HPLC purification of [^{77}Br]Br-WC-DZ (Column: Agilent SB-C18 250×9.4 mm: Mobile phase:19.5 % acetonitrile/80.5 % water/0.1 % TFA; Flow rate: 4 mL/min and UV: 250 nm; Blue: UV/Red: Radioactivity). [^{77}Br]Br-WC-DZ was collected at 21-23 min.

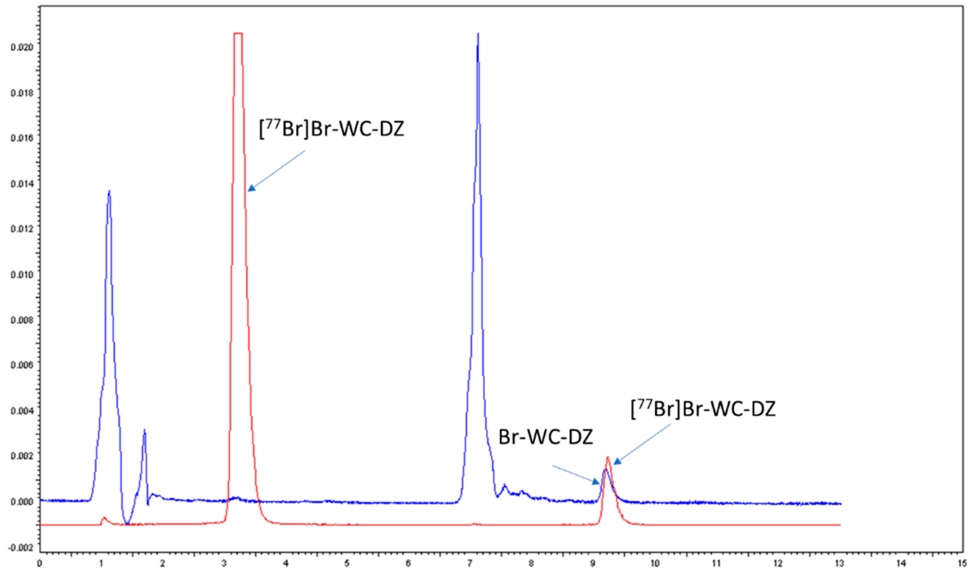

Figure S2. Analytical radio-HPLC of [^{77}Br]Br-WC-DZ (Column: Altima C18 250×4.6 mm: Mobile phase:30 % acetonitrile/70 % watter/0.1 % TFA; Flow rate: 2 mL/min and UV: 250 nm; Blue: UV/Red: Radioactivity), showing [^{77}Br]Br-WC-DZ at 3.3 min and a separate co-injection of the Br-WC-DZ standard and [^{77}Br]Br-WC-DZ demonstrating co-elution at 9.2 min. Note that this single chromatogram represents two different injections.

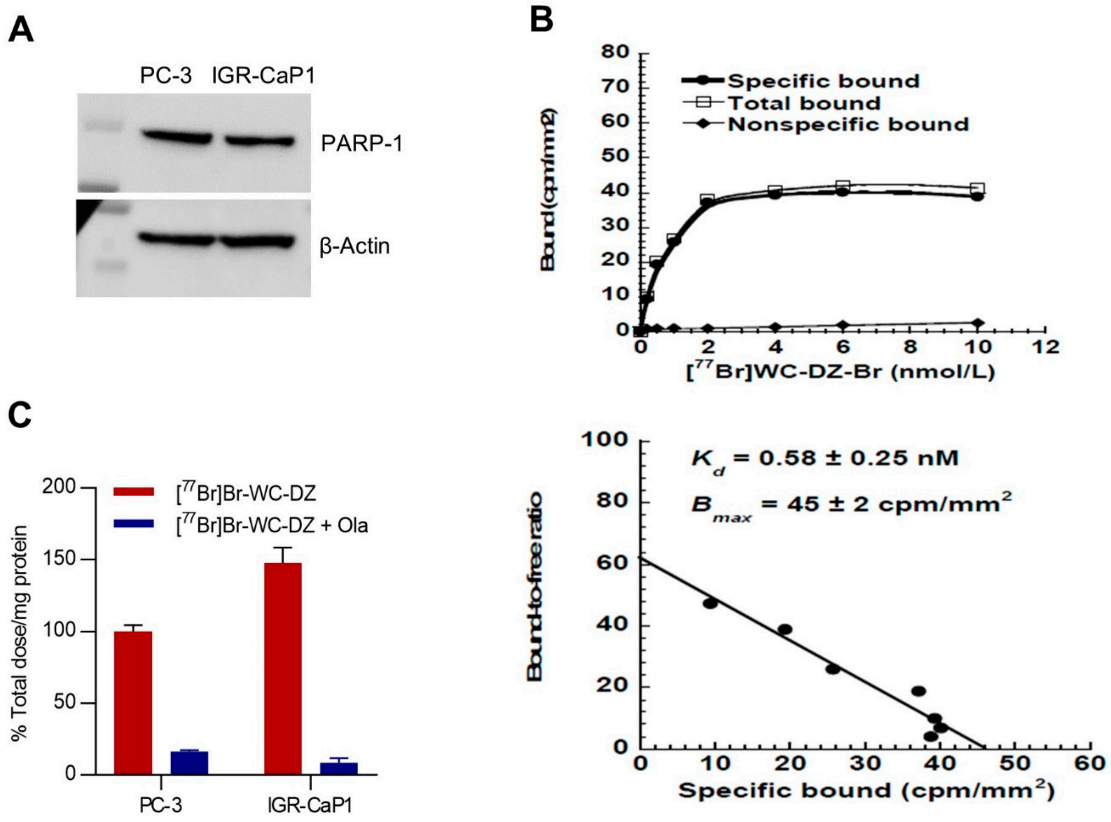

Figure S3A: Uncropped Western blot Images

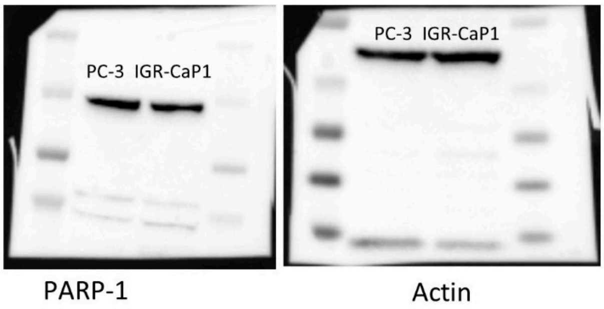

Figure S3. A, PARP-1 expression in prostate cancer cell lines PC-3 and IGR-CaP1 analyzed by Immunoblotting. β-Actin was used as a loading control. **B**, Binding affinity assay of [^{77}Br]Br-WC-DZ in PC-3 tumor xenograft. **C,** Cellular uptake of [^{77}Br]Br-WC-DZ in IGR-CaP1 and PC-3 cell lines with and without Olaparib (50 μM) blocking.

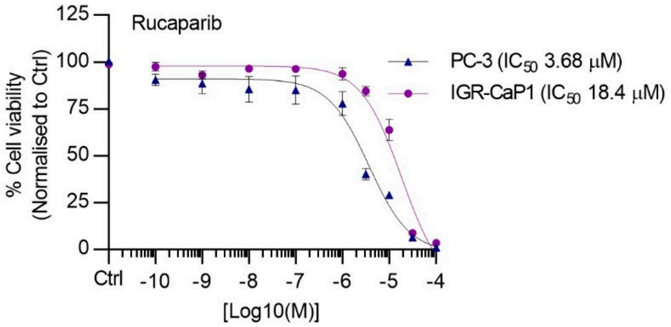

Figure S4. Effect of rucaparib in the viability of prostate cancer cell lines. PC-3 and IGR -CaP1 cells were treated with increasing doses of rucaparib (10-10 to 10-4 M) in biological replicates of five for 120 h. Cell viability was determined using MTS assay. Graph represents percentage (%) mean cell viability normalized to control ± SD.

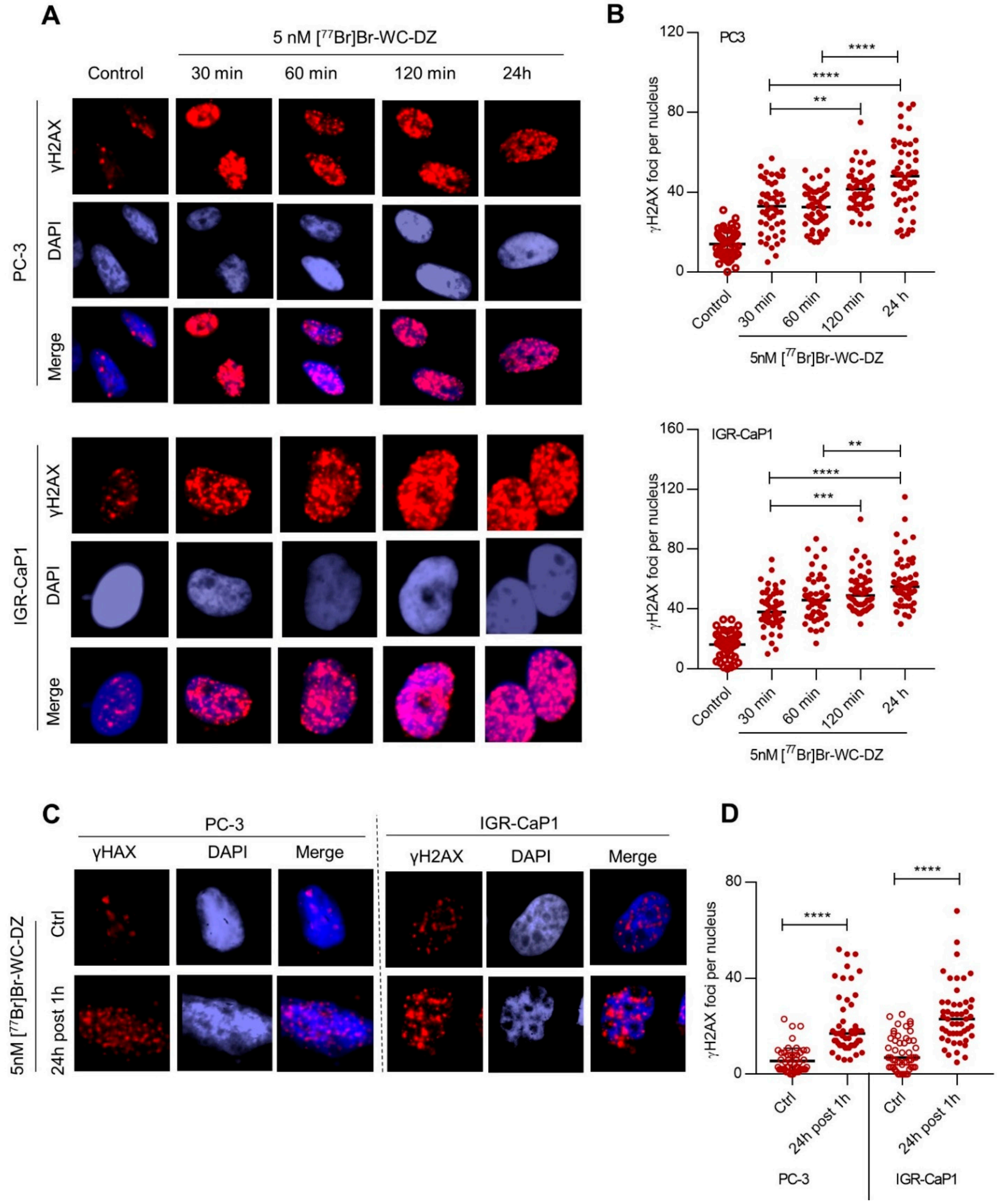

Figure S5. A, Immunofluorescence of γH2AX in PC-3 and IGR-CaP1 cells after treatment with [77Br]Br-WC-DZ (5 nM) for 30,60,120 min and 24h. **B,** γH2Ax foci counts per nucleus in the cells treated with [77Br]Br-WC-DZ. **C-D,** Residual γH2AX foci in PC-3 and IGR-CaP1 cells exposed to [77Br]Br-WC-DZ. Cells were treated with [77Br]Br-WC-DZ for 60 min, washed and γH2AX foci immunofluorescence staining was done after a 24 h recovery period. γH2Ax foci were counted on at least 50 cells per treatment, and results are depicted as dot plot distribution values (the median is also reported for each sample). ****, P < 0.0001; ***, P < 0.001; **, P < 0.01

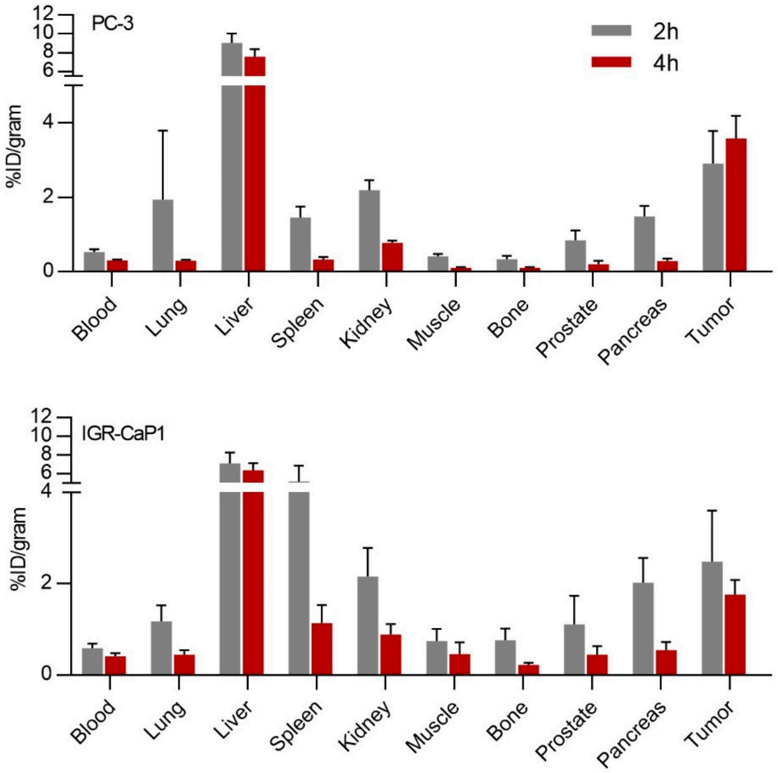

Figure S6. Biodistribution of [^{77}Br]Br-WC-DZ in prostate tumor bearing mice. Biodistribution of [^{77}Br]Br-WC-DZ (370 kBq) in blood and selected organs at 2h and 4h in athymic nude mice (n = 5) bearing PC-3 and IGR-CaP1 tumors.

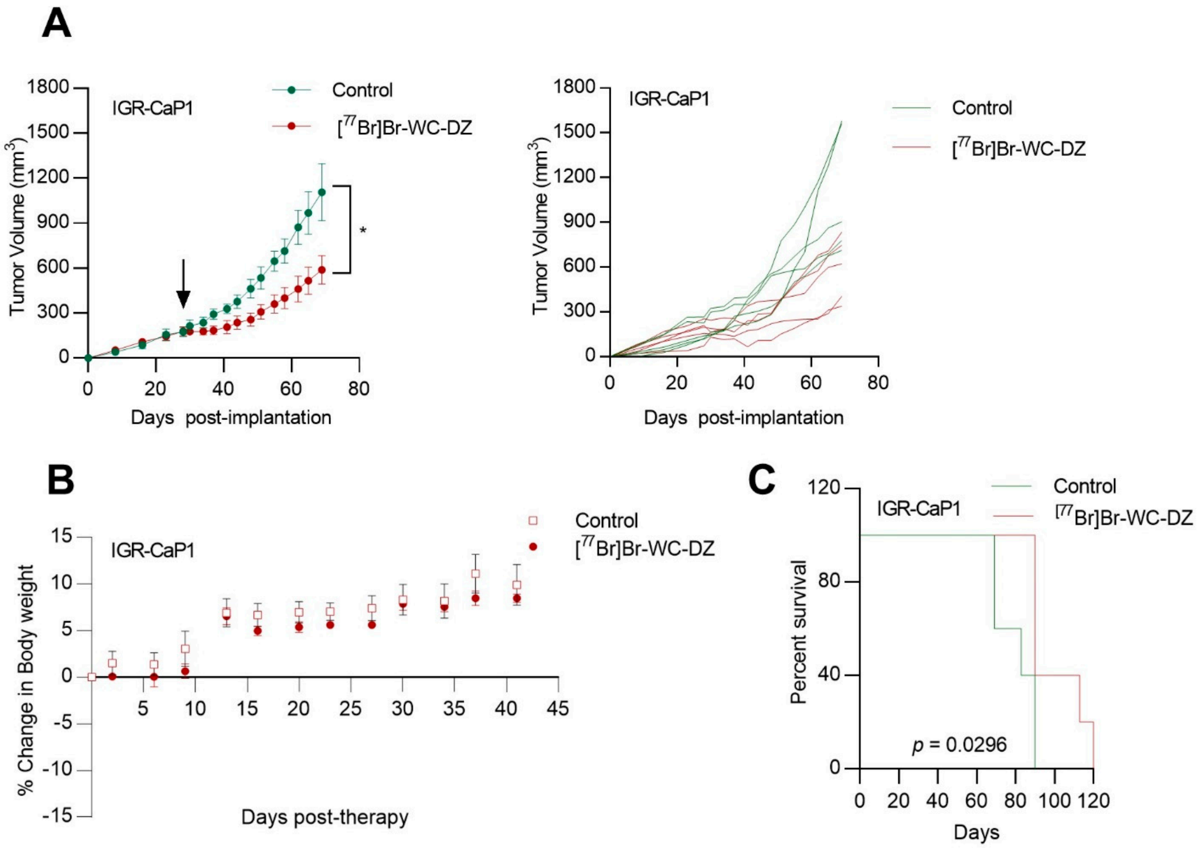

Figure S7. *In vivo* efficiacy of [^{77}Br]Br-WC-DZ in IGR-CaP1 tumor xenograft. **A,** Mean tumor volume (±SEM) in athymic nude mice (n = 5) after treatment with 56 MBq of [^{77}Br]Br-WC-DZ. Control group received saline. [^{77}Br]Br-WC-DZ significantly suppressed the growth of IGR-CaP1 ($p = 0.0296$) xenograft tumor growth compared to the vehicle. Individual tumor growth curves for control and [^{77}Br]Br-WC-DZ treated groups are also shown. **B,** The plots showing the average percent change in body weight of mice bearing IGR-CaP1 xenografts treated with saline (control) or [^{77}Br]Br-WC-DZ . **C,** Kaplan–Meier survival study of IGR-CaP1 tumor implanted mice showed improved survival of [^{77}Br]Br-WC-DZ treated mice compared to control. Statistical significance was determined using a Mantel–Cox log-rank test.

References

1. Reilly, S.W.; Makvandi, M.; Xu, K.; Mach, R.H. Rapid Cu-Catalyzed [211At]Astatination and [125I]Iodination of Boronic Esters at Room Temperature. *Org. Lett.* **2018**, *20*, 1752–1755, doi:10.1021/ACS.ORGLETT.8B00232.
